# Mental comorbidities in adolescents and young adults with juvenile idiopathic arthritis: an analysis of German nationwide health insurance data

**DOI:** 10.1186/s12969-023-00948-y

**Published:** 2024-01-05

**Authors:** Florian Milatz, Katinka Albrecht, Kirsten Minden, Ursula Marschall, Jens Klotsche, Johanna Callhoff

**Affiliations:** 1 Programme area Epidemiology and Health Services Research, Deutsches Rheuma-Forschungszentrum, a Leibniz Institute, Charitéplatz 1, 10117 Berlin, Germany; 2 Department of Pediatric Respiratory Medicine, Immunology and Critical Care Medicine, Charité – Universitätsmedizin Berlin, corporate member of Freie Universität Berlin and Humboldt-Universität zu Berlin, Berlin, Germany; 3 Department Medicine and Health Services Research, BARMER Institute for Health System Research, Wuppertal, Germany; 4 Institute for Social Medicine, Epidemiology and Health Economics, Charité – Universitätsmedizin Berlin, corporate member of Freie Universität Berlin and Humboldt- Universität zu Berlin, all Germany, Berlin, Germany

**Keywords:** Depression, Anxiety, Mental comorbidities, Adolescents, Health insurance data, Juvenile idiopathic arthritis

## Abstract

**Background:**

Studies on prevalence rates of mental comorbidities in patients with juvenile idiopathic arthritis (JIA) have reported varying results and provided limited information on related drugs. The purpose of this study was to determine the prevalence of selected mental health diagnoses and the range of associated drug prescriptions among adolescents and young adults (AYA) with JIA compared with general population controls.

**Findings:**

Nationwide statutory health insurance data of the years 2020 and 2021 were used. Individuals aged 12 to 20 years with an ICD-10-GM diagnosis of JIA in ≥ 2quarters, treated with disease-modifying antirheumatic drugs and/or glucocorticoids were included. The frequency of selected mental health diagnoses (depression, anxiety, emotional and adjustment disorders) was determined and compared with age- and sex-matched controls. Antirheumatic, psychopharmacologic, psychiatric, and psychotherapeutic therapies were identified by Anatomical Therapeutic Chemical (ATC) codes and specialty numbers. Based on data from 628 AYA with JIA and 6270 controls, 15.3% vs. 8.2% had a diagnosed mental health condition, with 68% vs. 65% receiving related drugs and/or psychotherapy. In both groups, depression diagnosis became more common in older teenagers, whereas emotional disorders declined. Females with and without JIA were more likely to have a mental health diagnosis than males. Among AYA with any psychiatric diagnosis, 5.2% (JIA) vs. 7.0% (controls) received psycholeptics, and 25% vs. 27.3% psychoanaleptics.

**Conclusions:**

Selected mental health conditions among 12-20-year-old JIA patients are diagnosed more frequently compared to general population. They tend to occur more frequently among females and later in childhood. They are treated similarly among AYA regardless of the presence of JIA.

**Supplementary Information:**

The online version contains supplementary material available at 10.1186/s12969-023-00948-y.

## Background

Juvenile idiopathic arthritis (JIA) is the most common chronic rheumatic disease in pediatrics describing a heterogeneous group of inflammatory rheumatic diseases of unknown origin [1]. Affected individuals typically suffer from acute pain and swelling, but also from joint damage and comorbid conditions that might develop during the course of their disease [2]. Resulting limitations in daily life, time-consuming physical therapies, and medication side effects can fuel dissatisfaction and psychological distress, which in turn detoriates disease self-management and treatment adherence [3–5]. Typical issues faced by adolescents are therefore compounded by the challenge of managing their chronic disease [6].

Previous studies among adolescents with JIA have found an increased risk for mental health impairments; however, rates of symptoms and diagnoses have varied widely [3, 7–10]. Most of the existing studies focused on mental health issues using self-report questionnaires and from a quality of life perspective. Their statements were partly limited by small sample sizes, lack of controls or missing information on related drug prescriptions [3].

In this study, we aimed to use health insurance data to determine the prevalence of diagnosed selected mental health conditions and their medication and psychotherapeutic treatment in AYA with JIA compared to a control group from the general population.

## Findings

### Methods

Cross-sectional BARMER claims data collected in the years 2020 and 2021 were used. The BARMER statutory health insurance fund is one of the largest health insurance companies in Germany and covers around 660,000 adolescents aged 12-20 years, corresponding to around 11% of all juvenile inhabitants of this age group with a statutory health insurance [11]. Germany has health care coverage for all permanent residents, allowing them to have access to the statutory health insurance system. There is no co-payment for treatment for people under 18 years of age and low co-payment rate for adults (e.g. up to 10 euros for a prescription for medication).

Inclusion criteria were as follows: 1) age between 12 to 20 years, 2) continuous insurance in 2020 and 2021, 3) at least one International Statistical Classification of Diseases German Modification (ICD-10-GM) code for JIA in at least two quarters of 2020 or 2021, 4) at least one of the following ICD-10-GM codes present before the age of 16 years: Juvenile arthritis (M08.X, M09.0 and L40.5†) and/or M45.0, M46.0, M46.8, M46.9, M07.0-3/L40.5, M05.X, M06.0, M06.1), and 5) at least one prescription for a disease-modifying antirheumatic drug (DMARD) or systemic glucocorticoid therapy in order to increase the reliability of diagnosis.

JIA was categorized into the following categories: polyarthritis, adult type (M08.0), enthesitis-related arthritis/juvenile spondyloarthritis (M08.1), systemic JIA (M08.2), RF- polyarthritis (M08.3), oligoarthritis (M08.4), psoriatic arthritis (M09.0) and undifferentiated JIA (M08.8, M08.9). When conflicting diagnoses were recorded in the same individual, only one diagnosis was assigned using the following hierarchy: M08.0, M08.1, M08.2, M08.3, M09.0, M08.4, M08.8/M08.9. If e.g. someone had an ICD-10-GM diagnosis of both M08.1 and M08.8 in their data, they were assigned to the enthesitis-related arthritis/juvenile spondyloarthritis (M08.1) group.

For each AYA with JIA we matched 10 controls with the same sex and age. Controls were randomly selected in the population without any condition of JIA in the complete years 2020 or 2021 as defined in the inclusion criteria. Controls were eligible independent of how often they visited a doctor’s office in 2020 or 2021 (it was possible that controls had no visits).

#### (Anti)rheumatic therapies and care

Antirheumatic therapies were identified using Anatomical Therapeutic Chemical (ATC) codes and included conventional synthetic (cs)DMARDs (azathioprine, cyclophosphamide, chloroquine, hydroxychloroquine, leflunomide, methotrexate, mycophenolate, sulfasalazine), biologic (b)DMARDs (abatacept, adalimumab, anakinra, canakinumab, certolizumab, etanercept, golimumab, infliximab, rituximab, sarilumab, secukinumab, tocilizumab), targeted synthetic (ts)DMARDs (baricitinib, tofacitinib), nonsteroidal antirheumatic drugs (NSAIDs) and systemic glucocorticoids (GCs). Rheumatology care was identified by specific billing codes for juvenile and adult rheumatology and by a specialist physician number for adult internal rheumatology.

#### Diagnoses and therapy of psychological morbidities

The following diagnoses were identified by ICD-10-GM codes: depression (F32, F33, F34, F38), anxiety disorders (F40,41), emotional disorders (F92, F93) and adjustment disorders (F43). Prescribed drugs related to psychological disorders include psycholeptics (N05), such as antipsychotics (N05A), anxiolytics (N05B), sedatives (N05C) as well as homeopathic psycholeptics (N05H), and psychoanaleptics (N06), such as antidepressants (N06A), non-selective monoamine reuptake inhibitors (N06AA) and selective serotonin reuptake inhibitors (N06AB). All drugs prescribed at least once in 2020 or 2021 are reported. Psychological/psychiatric therapy was identified by physician specialist numbers. The Defined Daily Doses (DDDs) dispensed are reported, providing information on the assumed average maintenance dose per day for a drug used for its main indication [12]. All ICD-10-GM, ATC-codes, physician specialist numbers and billing codes are reported in Suppl. Table S1.

#### Statistical analysis

Results are provided for AYA with JIA and controls, stratified by sex and age groups: 12-14, 15-17, and 18-20 years. In order to exclude accidental identifiability or inferences to individuals, no data is presented in groups with a case number <30. The frequencies among groups were compared using a Chi-square-Test as appropriate. As part of a sensitivity analysis, we also determined the frequency of psychological disorders based on data from 2019. The short report was written in accordance with the REporting of studies Conducted using Observational Routinely-collected health Data (RECORD) Statement [13].

## Results

**Fig. 1:**
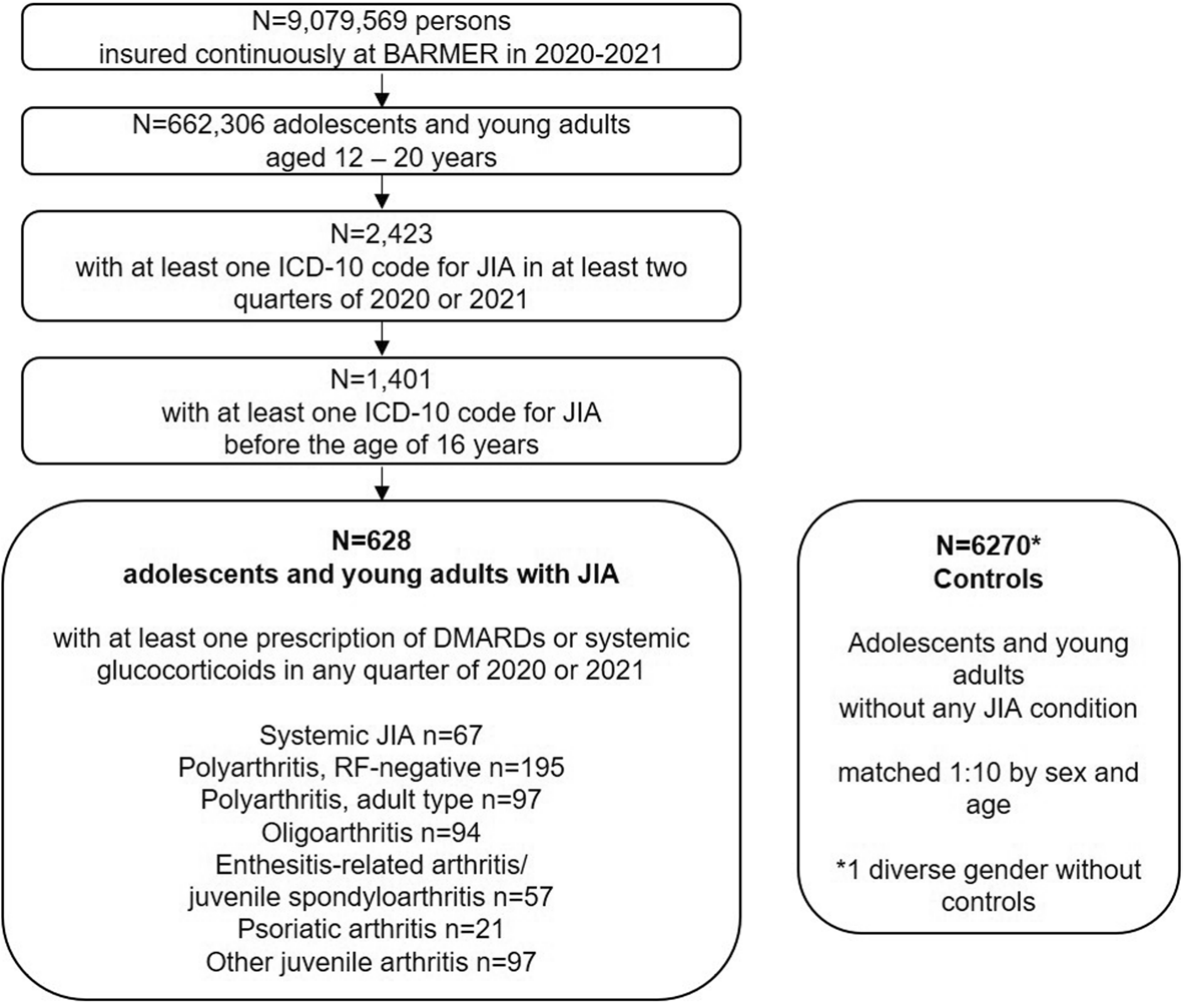
Flow Diagram of selected adolescents and young adults

In total, 628 AYA with JIA and 6,270 age- and sex-matched controls were included in the study. One of the AYA with JIA is of “diverse” gender, for this person we did not find matched controls. A flow diagram is presented in Fig. 1.

Of the 88% of AYA with JIA receiving DMARD therapy, about 48% received a TNF-inhibitor and 50% methotrexate. Female adolescents more often had csDMARDs (f 54% vs. m 45%) and NSAIDs (49% vs. 41%), while males were more frequently prescribed bDMARDs (f 54% vs. m 63%). Systemic glucocorticoids and bDMARDs were increasingly used with higher ages, while methotrexate was more often used in younger adolescents. bDMARD therapy was most frequent in systemic JIA and polyarthritis. More information on the characteristics and antirheumatic therapy is presented in Table 1.

**Table 1:** Characteristics of adolescents and young adults with JIA

**Variable**	**JIA**			
	**total**	**12-14 years**	**15-17 years**	**18-20 years**
	(*n*=628)	(*n*=182)	(*n*=234)	(*n*=212)
**Sociodemographic data**				
Age, years, mean (SD)	16.1 (2.5)	13.0 (0.8)	15.9 (0.8)	19.0 (0.9)
Female, no. (%)	446 (71)	130 (71)	160 (68)	156 (74)
**JIA category, no. (%)**				
Polyarthritis, adult type	97 (15)	21 (11)	26 (11)	50 (24)
Polyarthritis, RF-negative	195 (31)	68 (37)	71 (30)	56 (26)
Systemic JIA	67 (11)	16 (8.8)	29 (12)	22 (10)
Oligoarthritis	94 (15)	37 (20)	36 (15)	21 (9.9)
Psoriatic arthritis	21 (3)	5 (2.7)	8 (3.4)	8 (3.8)
Enthesitis-related arthritis/juvenile spondyloarthritis	57 (9)	8 (4.4)	29 (12)	20 (9.4)
Other JIA	97 (15)	27 (15)	35 (15)	35 (16)
**Antirheumatic therapy, no. (%)**				
Any b/cs/tsDMARD	554 (88)	165 (91)	197 (84)	192 (91)
Any bDMARD	371 (59)	99 (54)	129 (55)	143 (67)
Any csDMARD	321 (51)	111 (61)	122 (52)	88 (41)
Any tsDMARD	9 (1.4)	3 (1.6)	4 (1.7)	2 (0.9)
Any NSAID	294 (47)	79 (43)	118 (50)	97 (46)
Systemic GCs	190 (30)	41 (22)	79 (34)	70 (33)
GCs monotherapy	74 (12)	17 (9)	37 (16)	20 (9.4)
**Rheumatology care, no (%)**				
Total	416 (66)	119 (65)	142 (61)	155 (73)
Pediatric	297 (47)	118 (65)	133 (57)	46 (22)
Adult	172 (27)	8 (4.4)	30 (13)	134 (63)

*JIA* Juvenile idiopathic Arthritis, *RF-* Rheumatoid factor-negative, *DMARD* Disease-modifying antirheumatic drug, *bDMARD* Biological DMARD, *csDMARD* Conventional synthetic DMARD, *tsDMARD* Targeted synthetic DMARD, *NSAIDs* Non-steroidal anti-inflammatory drugs, *GCs* Glucocorticoids, *SD* Standard deviation

### Psychological disorders and related drug prescription

**Fig. 2:**
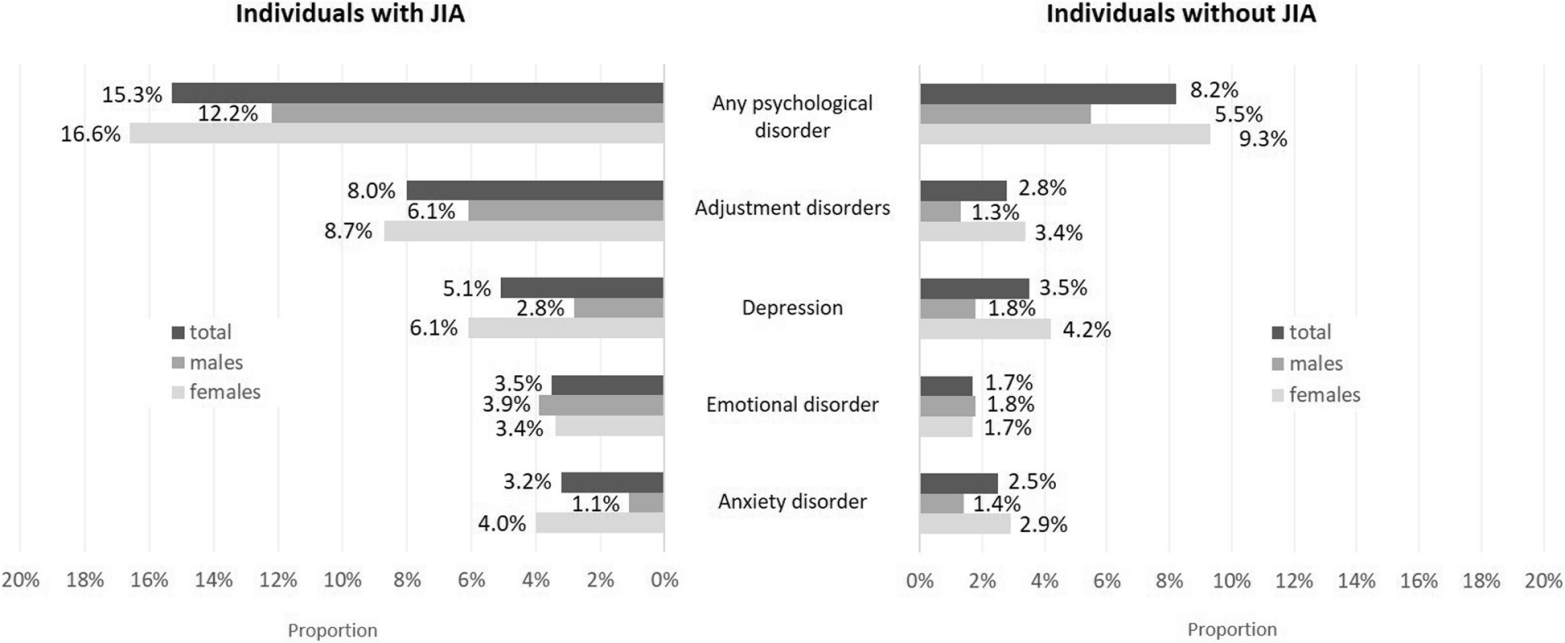
Prevalence of selected psychological disorders among females and males with and without JIA

Among 628 AYA with JIA, 15.3% (*n*=96) had any of the selected psychological diagnoses. In comparison, 8.2% (*n*=513) of controls were found to have a psychological diagnosis. In 2019, 16.0% of 506 patients and 7.3% of 5050 controls had one of these diagnoses. As shown in Fig. 2 adjustment disorders were diagnosed most frequently (JIA vs. controls: 8.0% vs. 2.8%, p(Chi-square) <0.001) followed by depression (5.1% vs. 3.5%, p(Chi-square) =0.04), emotional disorders (3.5% vs. 1.7%, p(Chi-square) =0.02), and anxiety disorders (3.2% vs. 2.5%, p(Chi-square) =0.29).

**Fig. 3:**
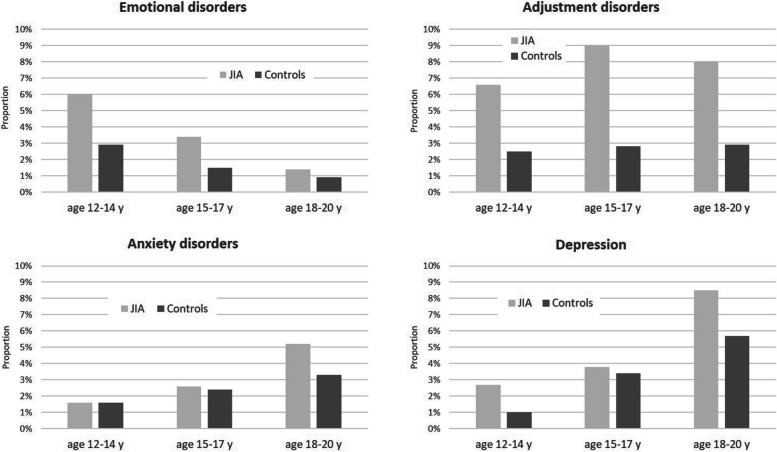
Prevalence of selected psychological diagnoses among individuals with (12-14 y, *n*=182; 15-17 y, *n*=234; 18-20 y, *n*=212) and without (12-14 y, *n*=1820; 15-17 y, *n*=2340; 18-20 y, *n*=2110) JIA by age group

**Fig. 4:**
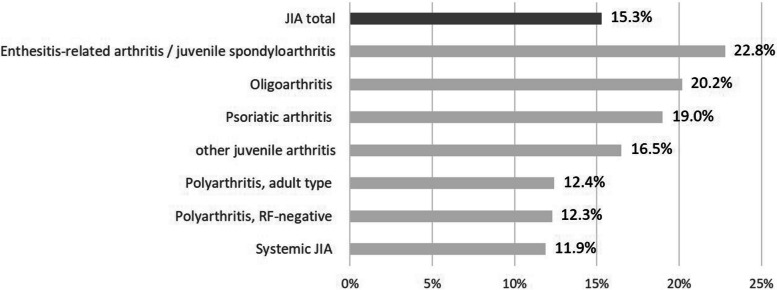
Proportion within each JIA category diagnosed with psychological disorder

Depression and anxiety were most common among individuals aged 18 to 20 year old among both JIA and control groups, whereas emotional disorders were diagnosed primarily among 12- to 14-year-olds (Fig. 3). Female patients were more likely to have depression than males. A psychiatric diagnosis was found more frequently in patients with oligoarthritis, enthesitis-related arthritis, or psoriatic arthritis than in patients with systemic JIA or polyarthritis (Fig. 4). More details on characteristics of AYA with and without a psychological diagnosis are shown in Table 2.

**Table 2:** Characteristics of individuals with and without JIA stratified by presence of any psychological disorder

**Variable**	**JIA**			**Controls**	
	Total (*n*=628)	any psychological disorders	no psychological disorders	any psychological disorders	no psychological disorders
		(*n*=96)	(*n*=532)	(*n*=513)	(*n*=5,757)
**Sociodemographic data**					
Age, years, mean (SD)	16.1 (2.5)	16.3 (2.6)	16.1 (2.5)	16.5 (2.5)	16.1 (2.5)
Female, no. (%)	446 (71)	74 (77.1)	372 (69.9)	414 (81)	4046 (70)
**JIA category, no. (%)**					
Polyarthritis, adult type	97 (15)	12 (12)	85 (16)	-	-
Polyarthritis, RF-negative	195 (31)	24 (25)	171 (32)	-	-
Systemic JIA	67 (11)	8 (8.3)	59 (11)	-	-
Oligoarthritis	94 (15)	19 (20)	75 (14)	-	-
Psoriatic arthritis	21 (3.3)	4 (4.2)	17 (3.2)	-	-
Enthesitis-related arthritis/juvenile spondyloarthritis	57 (9.1)	13 (13)	44 (8.3)	-	-
Other juvenile arthritis	97 (15)	16 (16)	81 (15)	-	-
**Antirheumatic therapy, no. (%)**					
Any bDMARD	371 (59)	62 (65)	309 (58)	1 (0.2)	6 (0.1)
Any csDMARD	321 (51)	45 (47)	276 (52)	1 (0.2)	1 (0.0)
Any tsDMARD	9 (1.4)	0 (0)	9 (1.9)	1 (0.2)	0 (0.0)
Any NSAID	294 (47)	55 (57)	239 (45)	92 (18)	712 (12)
Systemic GCs	190 (30)	36 (37)	154 (29)	12 (2.3)	111 (1.9)
GCs monotherapy	74 (11.8)	10 (10)	64 (12)	12 (2.3)	110 (1.9)
**Therapy related to psych. disorder, no. (%)**					
Psycholeptics (N05)	9 (1.4)	5 (5.2)	4 (0.8)	36 (7.0)	33 (0.6)
Antipsychotics (N05A)	2 (0.3)	2 (2.1)	0 (0)	28 (5.5)	20 (0.3)
Anxiolytics (N05B)	0 (0)	0 (0)	0 (0)	4 (0.8)	9 (0.2)
Sedatives (N05C)	7 (1.1)	3 (3.1)	4 (0.8)	16 (3.1)	6 (0.1)
Homeopathic psycholeptics (N05H)		0 (0)	0 (0)	1 (0.2)	0 (0.0)
Psychoanaleptics (N06)	40 (6.4)	24 (25)	16 (3.0)	140 (27)	118 (2.0)
Antidepressants (N06A)	25 (3.9)	20 (21)	5 (0.9)	106 (21)	40 (0.7)
Non-selective monoamine reuptake inhibitors (N06AA)	9 (1.4)	6 (6.3)	3 (0.5)	14 (2.7)	16 (0.3)
Selective serotonin reuptake inhibitors (N06AB)	15 (2.4)	13 (13)	2 (0.2)	83 (16)	18 (0.3)
Pediatric psychotherapy	71 (11)	49 (51)	22 (4.1)	241 (47)	96 (1.7)
Pediatric psychiatry	37 (5.9)	18 (19)	19 (3.6)	158 (31)	160 (2.8)
Drugs (N05/N06) or psychotherapy	105 (17)	65 (68)	40 (7.5)	331 (64)	220 (3.8)

*JIA* Juvenile idiopathic Arthritis, *RF*- Rheumatoid factor-negative, *RF+* Rheumatoid factor-positive, *DMARD* Disease modifying antirheumatic drug, *bDMARD* Biological DMARD, *csDMARD* Conventional synthetic DMARD, *tsDMARD* targeted synthetic DMARD, *NSAIDs* Non-steroidal antiinflammatory drugs, *GCs* Glucocorticoids, *SD* Standard deviation

Taking into account all persons who either had a diagnosed mental disorder or were being treated with related drugs or psychotherapy, 16.7% of AYA with JIA and 8.8% of controls were affected. About 7% of AYA with JIA had been diagnosed with depression or were taking an antidepressant.Among AYA with JIA diagnosed with any psychological disorder (*n*=96), 68% received related drugs and/or psychotherapy. This was also the case for a comparable proportion of controls diagnosed with any psychological disorder (65%). Psychotherapy, psychoanaleptics, and psycholeptics were documented with comparable frequency in the JIA and control group, whereas controls were more likely to have a prescription for psychiatric therapy (Table 2). Psychoanaleptics, especially antidepressants, and psycholeptics were only prescribed from the age of 15 and most frequently used in those aged 18 to 20 years. While mean DDDs for NSAIDs were similar in AYA with (101) and without (101) any psychological disorder, mean DDDs for GCs were lower (80) in AYA with than in AYA without any psychological disorder (106).

## Discussion

To our knowledge, this study on mental comorbidities in pediatric JIA patients and controls is based on one of the largest samples analyzing a wide range of prescribed medications related to mental health problems using health insurance data. We found that AYA with JIA had a higher risk of a mental health diagnosis than the control population.

A few previous studies on psychiatric morbidity in JIA have also highlighted an increased risk in specific outcomes compared with controls [7, 14–17]. Although our findings are in line with these results, comparability is mostly limited due to methodological discrepancy. Small heterogeneous samples as well as differences in case assessment (symptoms vs. diagnoses) and/or disease durations/activities may explain why few previous studies have stated no increased risk of mental health issues in JIA patients compared to general population controls [18–20]. In our study, the prevalence rate of psychological diagnoses seemed to be higher among females than among males, both in AYA with and without JIA. These results are consistent with trends in the general population [21] and those previously reported in JIA [14, 17, 20, 22]. We have shown that prevalence rates of selected mental disorders vary with age, with depression and anxiety diagnoses becoming increasingly common. Thus, our findings confirm previous studies on the relationship between age and mental health in JIA [19, 22, 23]. In our study, individuals with oligoarthritis more frequently had mental health diagnoses than individuals with polyarthritis. Conversely to our findings, previous studies showing that patients with a polyarticular course are at higher risk for mental health impairments than patients from other categories [3, 22]. However, since factors such as JIA onset, disease duration, sex, and age can influence the frequency and type of mental disorders [14], comparability with previous studies is limited. We found that about two thirds of individuals with JIA and diagnosed mental disorder were undergoing psychotherapeutic or psychopharmacological treatment. The similar proportion of controls with psychological disorders receiving treatment shows that JIA patients seem to have the same chance to receive treatment for diagnosed mental health issues than controls.

### Limitations and strengths

Our study had several limitations, including not clinically validated diagnoses of JIA and mental disorders. Therefore, prescriptions of DMARDS and/or GC were included to increase diagnostic reliability of JIA. Specialist contact may be underestimated as specialists working in general practitioners’ offices or in outpatient settings within university hospitals are not identifiable as rheumatologists in the data. The higher prevalence of mental disorders in patients compared to controls could also be due to the fact that patients have regular medical encounters, can express mental problems and have easier access to psychological/psychiatric care. This may also have been the case during the corona pandemic (when patients still had regular medical encounters). In addition, the pandemic itself as remarkable stressor may also have played a role. In order to rule out the latter, a sensitivity analysis was carried out, which, however, came to comparable results based on data from the pre-corona period. The study was not designed to address drug-related associations with depression or other mental disorders.

Despite these limitations, our data cover a broad nationwide DMARD/glucocorticoid-treated JIA cohort, representing about 4.5% of the estimated JIA population in Germany [24]. Strengths of claims data include the possibility to identify sex- and age matched controls without arthritis diagnoses. A main advantage of the data source is the complete coverage of all prescribed drugs.

## Conclusion

Selected psychological disorders among 12- to 20-year-olds with JIA are diagnosed more frequently than in controls without JIA. They are diagnosed with varying frequency across the age range and JIA categories, and are more common in females than males. Mental health issues are treated proportionally equally in adolescents with JIA compared to controls.

### Supplementary Information

**Additional file 1.** 

## Data Availability

No additional data are available.

## Abbreviations

AYA: Adolescents and young adults
JIA: Juvenile idiopathic Arthritis
DMARDs: Disease-modifying antirheumatic drugs
ICD-10: International Statistical Classification of Diseases
ICD-10-GM: German modification of the ICD-10
ATC: Anatomical Therapeutic Chemical
NSAIDs: Non-steroidal anti-inflammatory drugs
GCs: Glucocorticoids
DDDs: Defined Daily Doses
