# Supplementary material for: Mental comorbidities in adolescents and young adults with juvenile idiopathic arthritis: an analysis of German nationwide health insurance data
Source: Pediatr Rheumatol Online J. 2024 Jan 5;22:10. doi: 10.1186/s12969-023-00948-y (PMC10768083; doi:10.1186/s12969-023-00948-y)
Supplement: Supplementary file 1 — Additional file 1. [file 12969_2023_948_MOESM1_ESM.docx]

| **Juvenile Idiopathic Arthritis categories** | **ICD-codes** |
| --- | --- |
| Systemic | M08.2-, M06.1 |
| oligoarticular | M08.4- |
| Seronegative polyarthritis | M08.3-, M06.0 |
| seropositive polyarthritis | M08.0-, M05.x |
| Enthesitis-related/juvenile spondyloarthritis | M08.1-, M45.X, M46.0, M46.8, M46.9 |
| Psoriatic arthritis | M09.0-*, L40.5+, M07.0-3 |
| Other | M08.8-, M08.9-, M06.2-9 |
| **Psychological disorders** |  |
| Depression | F32, 33, 34, F38 |
| Anxiety disorders | F41, F40 |
| Emotional disorders | F92, 93 |
| Adjustment disorders | F43 |
| **Medication** | **ATC-Codes** |
| Psycholeptics | N05 |
| Antipsychotics | N05A |
| Anxiolytics | N05B |
| Sedatives | N05C |
| Homeopathic psycholeptics | N05H |
| Psychoanaleptics | N06 |
| Antidepressants | N06A |
| Non-selective monoamine reuptake inhibitors | N06AA |
| Selective serotonin reuptake inhibitors | N06AB |
| **Antirheumatic medication** |  |
| Conventional synthetic DMARDs |  |
| Azathioprine | L04AX01 |
| Cyclophosphamide | L01AA01 |
| Chloroquine | P01BA01 |
| Hydroxychloroquine | P01BA02 |
| Leflunomide | L04AA13 |
| Methotrexate | L01BA01, L04AX03, M01CX01 |
| Mycophenolate | L04AA06 |
| Sulfasalazine | M01CX02 |
| Biologic DMARDs |  |
| Abatacept | L04AA24 |
| Adalimumab | L04AB04 |
| Anakinra | L04AC03 |
| Canakinumab | L04AC08 |
| Certolizumab | L04AB05 |
| Etanercept | L04AB01 |
| Golimumab | L04AB06 |
| Infliximab | L04AB02 |
| Rituximab | L01XC02 |
| Sarilumab | L04AC14 |
| Secukinumab | L04AC10 |
| Tocilizumab | L04AC07 |
| tsDMARDs |  |
| Baricitinib | L04AA37 |
| Tofacitinib | L04AA29 |
| NSAIDs | M01A |
| Systemic glucocorticoids | H02AB |
|  |  |
| **Specialist care** | **Physician number / EBM codes** |
| Pediatric rheumatology | 04550, 04551 |
| Adult rheumatology | 13690, 13691, 13692, 13700, 13701  Internal rheumatoloy physician number: 31 |
| Pediatric psychiatry and/ or psychotherapy | 47, 69 |
| Psychiatry and psychotherapy,  Psychotherapeutic physician, Psychological psychotherapist, Psychosomatic medicine and psychotherapy | 58, 60, 61, 68 |
| ATC Anatomical Therapeutic Chemical, ICD International Statistical Classification of Diseases, NSAIDs | |
